# “Who writes what?” Using written comments in team-based assessment to better understand medical student performance: a mixed-methods study

**DOI:** 10.1186/1472-6920-12-123

**Published:** 2012-12-18

**Authors:** Jonathan Samuel White, Nishan Sharma

**Affiliations:** 1 Department of Surgery, Faculty of Medicine & Dentistry, University of Alberta, 10240 Kingsway Avenue, Edmonton, AB T5H 3V9, Canada

**Keywords:** Written comments, Undergraduate, Assessment, Medical students, Clerkship, Mixed-methods, Qualitative, Clinical performance, Team

## Abstract

**Background:**

Observation of the performance of medical students in the clinical environment is a key part of assessment and learning. To date, few authors have examined written comments provided to students and considered what aspects of observed performance they represent. The aim of this study was to examine the quantity and quality of written comments provided to medical students by different assessors using a team-based model of assessment, and to determine the aspects of medical student performance on which different assessors provide comments.

**Methods:**

Medical students on a 7-week General Surgery & Anesthesiology clerkship received written comments on ‘Areas of Excellence’ and ‘Areas for Improvement’ from physicians, residents, nurses, patients, peers and administrators. Mixed-methods were used to analyze the quality and quantity of comments provided and to generate a conceptual framework of observed student performance.

**Results:**

1,068 assessors and 127 peers provided 2,988 written comments for 127 students, a median of 188 words per student divided into 26 “Areas of Excellence” and 5 “Areas for Improvement”. Physicians provided the most comments (918), followed by patients (692) and peers (586); administrators provided the fewest (91). The conceptual framework generated contained four major domains: ‘Student as Physician-in-Training’, ‘Student as Learner’, ‘Student as Team Member’, and ‘Student as Person.’

**Conclusions:**

A wide range of observed medical student performance is recorded in written comments provided by members of the surgical healthcare team. Different groups of assessors provide comments on different aspects of student performance, suggesting that comments provided from a single viewpoint may potentially under-represent or overlook some areas of student performance. We hope that the framework presented here can serve as a basis to better understand what medical students do every day, and how they are perceived by those with whom they work.

## Background

Observation of the performance of medical students in the clinical environment is a key part of assessment and learning. There is increasing recognition of the importance of interprofessional education in medical education [1], and of the need to assess the performance of medical professionals working as part of a larger interprofessional team [2,3]. Numerous tools have been designed to assess observed medical student performance [4,5]. The ideal assessment system should sample observations widely and systematically, and generate written or verbal comments describing observed performance (qualitative data) as well as numerical data on some form of ratings scale (quantitative data) [6].

Many traditional assessment tools employ a numerical ratings scale followed by a section for general comments (eg. “Comments on this Trainee”). Although written comments have been utilized in this way for many years, little research has focused on how these comments are generated and what they represent. The few studies which have considered the use of written comments in feedback suggest that they may contain useful information and may be able to improve performance [5,7,8]. Recently there has been increased interest in the use of written comments to strengthen existing assessment methods, especially in the assessment of professionalism [9-12]. There is evidence that teachers and learners may place more emphasis on written comments than numerical ratings [13], and that the observations recorded as written comments may be distinct from those associated with numerical ratings of academic success [14]. A number of authors have pointed out that physicians’ direct observations of their learners’ day-to-day clinical performance on the healthcare team may be limited, that their ratings and comments may be prone to indirect inference and positive bias [6,15-17], and that physicians sometimes provide students with global impressions on generalized behaviours instead of giving specific advice on how to improve [18-21].

In previous work, we established the feasibility and acceptability of a team-based assessment model in a surgery clerkship, in which student performance is observed by a range of assessors on the surgical healthcare team including physicians, residents, patients, nurses, peers and administrators [22,23]. This method of assessment utilizes both numerical ratings and written comments. It was the aim of this study to examine the quantity and quality of written comments provided to medical students by different assessors, and to determine on what aspects of medical student performance different assessors can observe and provide comments.

## Methods

**Figure 1:**
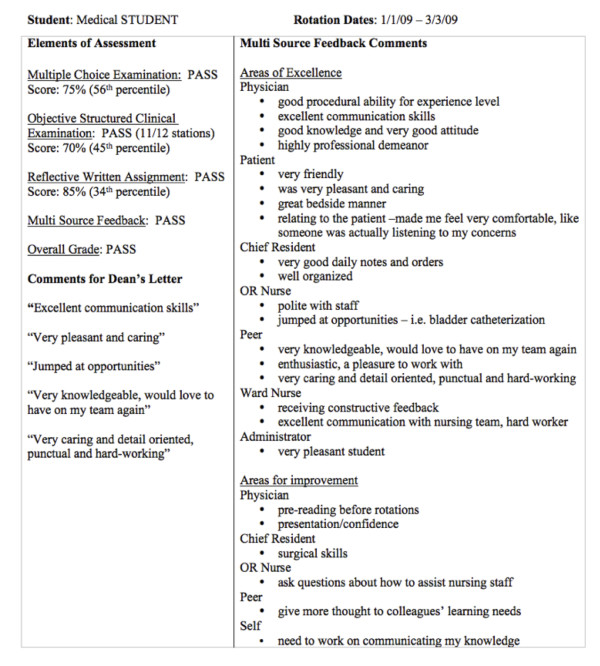
The “Summary of Assessment” form provided to students upon completion of the clerkship.

In the academic year 2009/10, the 7-week, Year 3 clerkship in General Surgery, Anesthesiology & Pain Medicine at our medical school adopted a team-based method of assessment, as previously described [22,23]. The assessment plan employed a multiple choice examination, an objective structured clinical examination, a reflective written assignment and the team-based assessment (TBA) of clinical performance. For the TBA element, each student had assessment forms completed by the following groups of assessors (number of forms in brackets): physician (surgeon) [6], physician (anesthesiologist) [2], chief resident [2], operating room nurse [2], patient [6], ward nurse manager on behalf of a team of ward nurses [2], peers (anonymous, 4–6) and administrators [1]. Forms were designed with the input of assessors and students, and contained areas for written comments on “Areas of Excellence” and “Areas of Improvement.” We chose to provide assessors with ‘cues’ in this way to avoid general or generic comments [24]. Each form also contained a number of items requiring a response required on a 5-point Likert scale, as previously described [23]. Forms were completed on paper, in person at the conclusion of a period in which the assessor was working with the student, with the exception of the peer forms (completed anonymously) and the administrator forms which were completed at the conclusion of the clerkship. Assessors were also provided with information and training about the new assessment method: posters were placed in prominent locations and information and advice was provided in person and online. Assessors were advised that providing written comments to students was encouraged but was not mandatory, and that the purpose of giving comments was to provide formative feedback. At the end of the clerkship, assessment forms were collected and all written comments were transcribed and entered into an electronic database. Each student was provided with a one-page “Summary of Assessment” listing all of the written comments given by each assessor type, shown in Figure 1[23].

**Figure 2:**
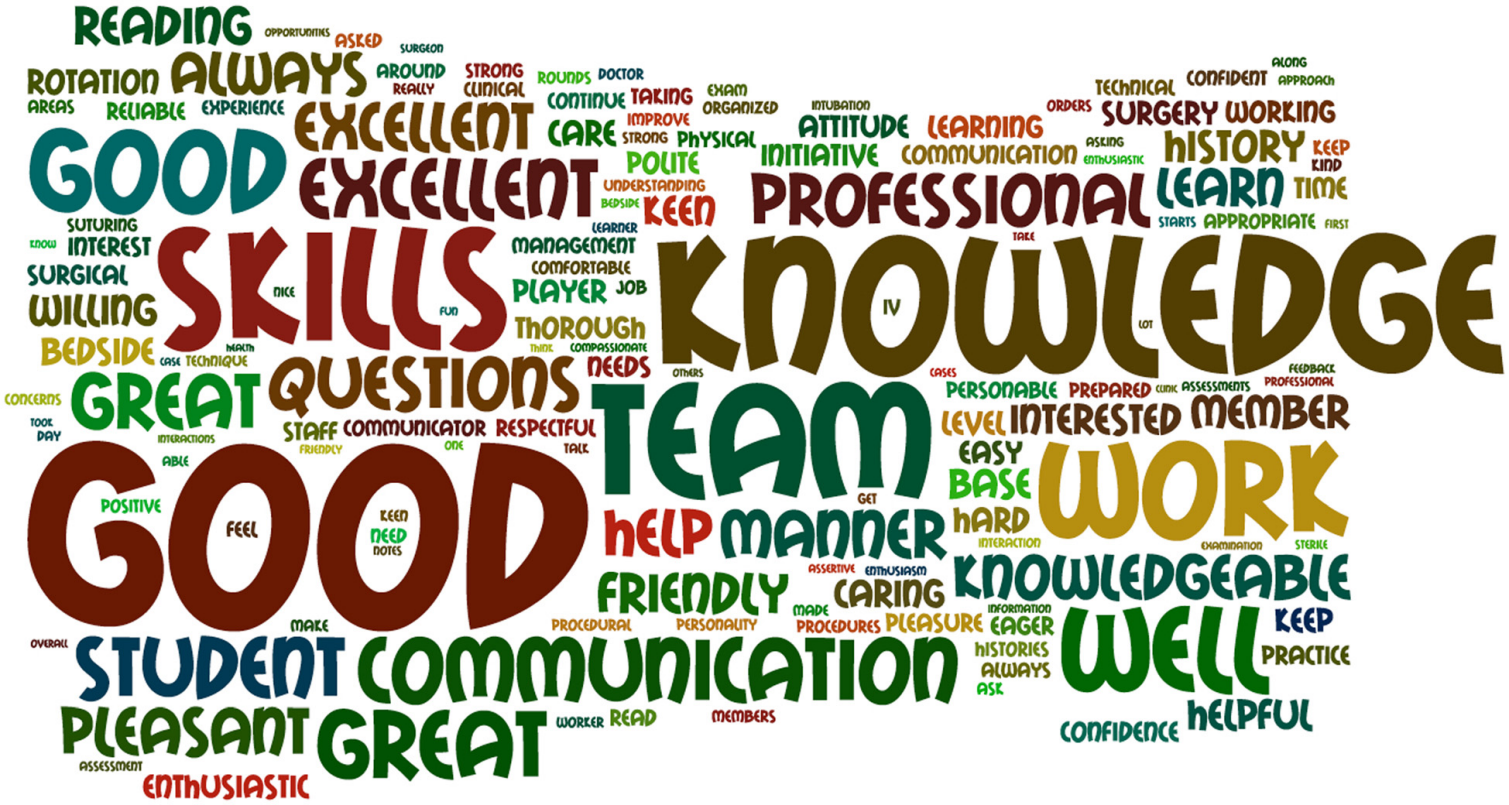
Example of a word cloud of written comments (all assessor groups combined).

A mixed-methods analysis was used to examine the written comments provided to students. Comments were anonymized before analysis, so that individual students and assessor could not be identified. The first step was a quantitative analysis, counting the number of comments and words provided to each student, broken down by assessor type and by “Excellence” versus “Improvement”. The second step was a thematic content analysis to review and categorize each comment, constructing a conceptual framework which described all of the comments provided [25]. This step was supplemented by the generation of “word clouds” for comments provided by each assessor group. This technique summarizes large amounts of data by presenting individual words in a ‘cloud’ in which the size of the word is directly related to its frequency of occurrence in the dataset (Figure 2). Two readers read all of the comments provided by each assessor group, and met weekly for 8 weeks to develop a framework which described the range of comments observed. The unit of analysis was not the comments given to the individual student, but rather the comments provided by each group of assessors. As far as possible, domains and sub-domains within the framework were named using direct quotation from assessor comments (*in situ* coding). Apart from comments from the administrator, each sub-domain represented at least two written comments from at least two assessors in the same group. As a wide range of behaviours was described in the written comments, multiple meetings were required to refine the coding structure and achieve consensus to ensure that the meaning of each domain and sub-domain was clear, and that each sub-domain was clearly separate from the others. Categorization and coding was continued until data saturation was achieved, and no further categories emerged. Once coding was completed, both readers read through the entire set of comments once again to ensure that no further domains or sub-domains could be identified. As a final step, the number of domains and sub-domains represented in the comments from each assessor group was also calculated. Approval for this study was granted by the University of Alberta’s Health Research Ethics Board (reference #8891).

## Results

### Quantitative analysis

87% of forms completed contained written comments. 1,068 assessors and 127 peers provided 2,988 written comments comprising a total of 22,183 words for 127 students.

116 students (91%) gave consent for their comments to be analyzed. 2,363 “Areas of Excellence” were noted and 625 “Areas for Improvement” (a ratio of 3.8:1). Each student received a median of 188 words of written feedback (range 91–330, interquartile range (IQR) 166–214) divided into 26 “Areas of Excellence” (range 13–37, IQR 23–28) and 5 “Areas for Improvement” (range 0–12, IQR 4–7). The median number of words per comment was 6 (range 4–10). Comments were distributed amongst assessor groups as shown in Table 1. Physicians provided the most comments (918), followed by patients (692) and peers (586); administrators provided the fewest (91). The ratio of “Excellence” to “Improvement” comments ranged from 9:1 (patients) to 1.8:1 (chief residents).

**Table 1:** Quantitative analysis of written comments provided by each assessor group

**Assessor group**	**# of assessors**	**Number of written comments**	**Ratio excellent: improvement**	**Areas of excellence comments per student median (range)**	**Areas for improvement comments per student median (range)**
Physician	145	918	710:208 (3.4)	6 (1–10)	2 (0–5)
Patient	764	692	567:63 (9.0)	5 (1–8)	0 (0–3)
Peers	127	586	487:99 (4.9)	4 (1–5)	1 (0–4)
Chief resident	17	282	219:125 (1.8)	2 (1–3)	1 (0–3)
Nurse: operating room	120	277	197:80 (2.5)	2 (0–2)	1 (0–2)
Nurse: ward / ward manager	21	142	112:30 (3.7)	1 (0–4)	0 (0–3)
Administrator	1	91	71:20 (3.6)	1 (0–1)	0 (0–1)
Total	1,195	2,988	2,363:625 (3.8)	26 (13–37)	5 (0–12)

### Qualitative analysis

The conceptual framework generated contained four major domains: ‘Student as Physician-in-Training’, ‘Student as Learner’, ‘Student as Team Member’, and ‘Student as Person’ (presented with representative quotations in Table 2). A total of 39 sub-domains were also identified. The domains and sub-domains represented in the comments from each assessor group is shown in Table 3.

**Table 2:** The conceptual framework of written comments provided on observed medical student performance (source for comments in brackets)

**Domain/Sub-domain**	**Representative quotations**
**PHYSICIAN-IN-TRAINING (14 sub domains)**	
Medical knowledge	Excellent knowledge of anatomy and surgical procedures (resident), Good knowledge of my problem (patient), very strong knowledge (physician)
Patient rapport	Established rapport with patients (physician)
Communication with patients - history taking	Very good focused patient histories (physician), Asked questions appropriately due to my condition (patient)
Communication with patients – listening	Paid attention to me and was genuinely listening to my concerns (patient)
Communication with patients – explaining	Explained things very well (patient)
Physical examination	Give more precise directions during physical examination (patient)
Organization of findings: information management	Organized well thought out assessment and plan with each patient (physician), Very organized, good notes (resident), Seemed really organized, was on top of what was going on with patients (peer), learning to be more organized (ward nurse)
Organization of findings: critical thinking	Logical, can reason through problems (physician), Critically thinking regarding plan of care (ward nurse)
Organization of findings: diagnosis & management	Keep developing your own treatment plan (resident)
Organization of findings: written notes	Writing accurate notes (ward nurse), writing notes (resident), more legible writing (peer)
Organization of findings: oral presentation	Focus on summarizing (physician)
Technical Skills: gloving/gowning	Practice gowning and gloving (OR nurse)
Technical Skills: asepsis	Very aware of sterile surroundings (OR nurse)
Technical Skills: procedures	Inserted a catheter with great skill (OR nurse), Practice more bag and ventilation, intubation (physician), Wonderful technical skills (peer), Removing staples (patient)
**LEARNER (8 sub domains)**	
Interest, enthusiasm	Demonstrated a keen interest in surgery (admin), Seemed interested in surgery (physician), Her enthusiasm and energy are an inspiration (peer)
Initiative & self-direction	Prepared early for rounds (peer), Showed initiative to ensure that orders were written appropriately (ward nurse), Appears highly self motivated (physician), Good initiative, came in early for rounds (resident)
Preparation for learning	Excellent preparation for OR and clinics (physician), Always prepared for OR (resident), Always well prepared for learning experiences (peer), He was prepared for surgeries (OR nurse)
Asking questions to learn	Actively looking for opportunities for experience (ward nurse), Keen to learn (peer)
Openness to feedback & self-improvement	Open for suggestion and criticism to do better (OR nurse), Took constructive criticism well and applied accordingly (physician)
Progress & improvement	Experience and practice will enhance her abilities (patient), Will improve appropriately with experience (physician)
Confidence	Continue to develop assertiveness and confidence (ward nurse), Speaks too soft. May need to work on confidence (physician), Very knowledgeable and confident in his abilities (peer), Sound more confident when you speak (patient)
Career suitability	Excellent student should be encouraged to do surgery (physician), well suited to anesthesia (physician), should do surgical specialty (resident), His personality is greatly suited for being a physician (patient)
**WORKING TEAM MEMBER (8 sub domains)**	
Work ethic	Always stuck around to make sure we had help (OR nurse), Works hard, interested (physician), Hardworking and always able to lend a hand (resident)
Organization/time management	Arrived late for clerkship session (admin), Always prepared with patient information (ward nurse), Very systematic (resident)
Conscientousness	Trustworthy, conscientous, reliable (resident), Conscientous and dependable (peer)
Helpfulness	Helpful member of the team (resident), Always more than willing to help out (peer), Very willing to help with patient transfer post surgery (OR nurse)
Team communication	Did not respond to page (admin), Conveyed information to all team members in a positive, informative manner (ward nurse), Did not introduce herself (ward nurse), Communicated effectively (resident)
Cooperation & participation	Very cooperative team player (OR nurse), Consistent positive interaction with all health care team members (ward nurse), Went out of her way to help the team (peer), Sometimes seemed focused on personal objectives over team (peer)
Follows instructions	Took suggestions and instructions comfortably (OR nurse),
Leadership	Take a leadership role as you have the skills to be a great leader (peer)
**PERSON (9 sub domains)**	
Compassion	Compassionate with patients (ward nurse), Caring, compassionate and involved (resident), Showed care and compassion for the patients he took care of (peer), Feeling for my pain (patient)
Patient-centredness	Very understanding to patient's needs (ward nurse), Was attentive to patient’s needs (physician), Advocated for patients well (resident), Seemed really interested about me (patient)
Personable	Pleasant, polite and cheerful student (admin), Approachable and friendly (OR nurse), Delightful (ward nurse), Pleasant to work with (physician), Very collegial and enjoyable to be around (resident),Easy to work with (peer), Was very friendly which made me feel comfortable (peer)
Respect for others	Treated me with respect (patient), Very diplomatic and respectful of others (peer), Timid and respectful (ward nurse), Very respectful with nurses and surgeon (OR nurse)
Humour	Kept things things entertaining, helped lighten our stress (peer), Sense of humor (patient)
Politeness	Polite and pleasant natured (patient), Aware of appropriate interactions (ward nurse), Was very polite and professional (OR nurse), Very polite and professional (admin)
Resilience	Handled stressful situations very well (peer)
Common sense	Good common sense (physician)
Honesty & integrity	Honest and professional (physician), clear and honest (patient)

**Table 3:** Domains and sub-domains of the conceptual framework represented in comments from each assessor group (‘Y’ represents the presence of at least two comments from at least two assessors coded in this sub-domain, one assessor in the case of administrator)

	**Physician**	**Resident**	**OR Nurse**	**Ward Nurse**	**Admin**	**Peer**	**Patient**
**PHYSICIAN-IN-TRAINING (14 sub domains)**							
Medical Knowledge	Y	Y	Y	Y	Y	Y	Y
Patient rapport	Y		Y			Y	Y
Communication with patients - history taking	Y	Y					Y
Communication with patients – listening							Y
Communication with patients – explaining							Y
Physical examination	Y	Y					Y
Organization of findings: information management	Y	Y		Y		Y	
Organization of findings: critical thinking	Y	Y		Y			
Organization of findings: diagnosis & management	Y	Y					
Organization of findings: written notes		Y		Y		Y	
Organization of findings: oral presentation	Y	Y					
Technical Skills: gloving/gowning			Y				
Technical Skills: asepsis			Y				
Technical Skills: procedures	Y	Y	Y			Y	Y
**LEARNER (8 sub domains)**							
Interest, enthusiasm	Y	Y	Y	Y	Y	Y	
Initiative & self-direction	Y	Y	Y	Y		Y	
Preparation for learning	Y	Y	Y	Y		Y	
Asking questions to learn	Y	Y	Y	Y		Y	
Openness to feedback & self-improvement	Y		Y	Y			
Progress & improvement	Y						Y
Confidence	Y	Y	Y	Y	Y	Y	Y
Career suitability	Y	Y					Y
**WORKING TEAM MEMBER (8 sub domains)**							
Work ethic	Y	Y	Y			Y	
Organization/time management	Y	Y		Y	Y	Y	
Conscientousness	Y	Y				Y	
Helpfulness	Y	Y	Y	Y		Y	
Team communication	Y	Y	Y	Y	Y	Y	
Cooperation		Y	Y	Y		Y	
Follows instructions			Y				
Leadership						Y	
**PERSON (9 sub domains)**							
Compassion		Y		Y		Y	Y
Patient-centredness	Y	Y		Y		Y	Y
Personable	Y	Y	Y	Y	Y	Y	Y
Respect for others			Y	Y		Y	Y
Humour						Y	Y
Politeness			Y	Y	Y		Y
Resilience						Y	
Common sense	Y						
Honesty & integrity	Y						Y

The domain ‘Student as Physician-in-Training’ was developed to describe comments relating to the behaviours often referred to as ‘clinical skills’, i.e. those skills involved in the day-to-day duties of “doctoring”. There were 14 sub-domains which described students’ general medical knowledge and rapport with patients, and students’ communication with patients (history taking, listening, explaining), physical examination, organization of findings (information management, critical thinking, diagnosis and management, written notes and oral presentation) and technical skills (gloving/gowning, asepsis, procedures). The number of sub-domains covered by each group was as follows: physician: 9, resident: 9, OR nurse: 5, ward nurse: 4, administrator: 1, peer: 5, patient: 7. Most assessor groups provided comments relating to medical knowledge, information management and procedural skills. Physicians and residents commented on critical thinking and oral presentation skills. Residents, peers and ward nurses commented on written notes. Only physicians, residents and patients commented on history-taking; only physicians and residents commented on skills relating to diagnosis and management; only OR nurses commented on aseptic technique and gowning/gloving. Only patients provided comments on listening and explaining.

The domain ‘Student as Learner’ was developed to describe comments relating primarily to the student’s attitudes and behaviour relating to learning. Eight sub-domains were identified including: interest/enthusiasm, initiative & self-direction, preparation for learning, asking questions to learn, openness to feedback & self-improvement, progress & improvement, confidence and career suitability. The number of sub-domains covered by each group was as follows: physician: 8, resident: 6, OR nurse: 6, ward nurse: 6, administrator: 2, peer: 5, patient: 3. The majority of assessor groups provided comments in the areas of interest/enthusiasm, initiative, preparation to learn and student confidence. Physicians and nurses commented on openness to feedback, suggesting they were providing feedback to students. Seven of the eight assessor groups commented on student confidence, but only physicians and patients commented on student progress and improvement (eg. ‘this student has improved/will improve with more experience’). Physicians, residents and patients commented on the student’s suitability for medicine in general or for a certain speciality in particular.

The domain ‘Student as Team Member’ was developed to describe comments relating directly to the student’s work within the healthcare team, and their interactions with other team members. Eight sub-domains were identified here, including: work ethic, organization/time management, conscientiousness, helpfulness, team communication, cooperation, follows instructions and leadership. The number of sub-domains covered by each group was as follows: physician: 5, resident: 6, OR nurse: 5, ward nurse: 4, administrator: 2, peer: 7, patient: 0. Student peers provided the most comments in this domain, covering 7 of the 8 sub-domains (omitting ‘follows instructions’), and were the only group to provide comments on ‘leadership’ (eg. ‘student could take a leadership role on the team’). Physicians provided no comments on cooperation with other team members, but these sub-domains were covered by comments from operating room nurses and ward nurses, who also commented on team communication. Administrators commented on organization/time management and team communication. Patients provided no comments at all in this domain.

The domain ‘Student as a Person’ was developed to describe comments relating specifically to students’ personal attributes. Nine sub-domains were identified, including: compassion, patient-centredness, personability, respect for others, humour, politeness, resilience, common sense and honesty/integrity. The number of sub-domains covered by each group was as follows: physician: 4, resident: 3, OR nurse: 3, ward nurse: 5, administrator: 2, peer: 6, patient: 7. All assessor groups commented on personability, and most groups commented on compassion, patient-centredness, respect for others and politeness. Only peers and patients commented on humour, only peers commented on resilience, and only physicians commented on common sense.

### Representation of domains by each assessor group

For each domain, the proportion of sub-domains represented in the written comments is presented in Table 4. Of the 39 possible sub-domains, the number covered by each group was as follows: physician: 26 (67%), resident: 24 (62%), peer: 23 (59%), OR nurse: 19 (49%), ward nurse: 19 (49%), patient: 17 (44%), administrator: 7 (18%).

**Table 4:** Number and proportion of sub-domains represented in the written comments of each assessor group (proportions in brackets)

	**Total sub-domains**	**Physician**	**Resident**	**OR Nurse**	**Ward Nurse**	**Admin**	**Peer**	**Patient**
Physician-in-Training	14	9	9	5	4	1	5	7
		(0.64)	(0.64)	(0.36)	(0.29)	(0.07)	(0.36)	(0.50)
Learner	8	8	6	6	6	2	5	3
		(1.00)	(0.75)	(0.75)	(0.75)	(0.25)	(0.63)	(0.38)
Team Member	8	5	6	5	4	2	7	0
		(0.63)	(0.75)	(0.63)	(0.50)	(0.25)	(0.88)	(0.00)
Person	9	4	3	3	5	2	6	7
		(0.44)	(0.33)	(0.33)	(0.56)	(0.22)	(0.67)	(0.78)
TOTAL	39	26	24	19	19	7	23	17
		(0.67)	(0.62)	(0.49)	(0.49)	(0.18)	(0.59)	(0.44)

Residents and physicians provided the most representation of “Physician-in-Training” (64% of sub-domains each), and physicians provided the most representation of “Learner” (100% of sub-domains). Peers and residents provided the most representation of “Team Member” (88% and 75% of sub-domains respectively), while patients and peers provided the most representation of “Person” (78% and 67% of sub-domains respectively).

## Discussion

This study demonstrates that a wide range of observed medical student performance is recorded in written comments provided by members of the surgical healthcare team. This study also demonstrates that different groups of assessors provide comments on different aspects of student performance, suggesting that comments provided from a single viewpoint may potentially under-represent or overlook some areas of student performance.

In their roles as teachers and expert clinicians, we suggest that physicians see students primarily as trainee doctors with a certain set of skills which they must learn. Thus, “Physician-in-Training” and “Learner” comprise over half of the sub-domains in the framework, and these domains are well-represented in the written comments provided by physicians. In contrast, physicians covered fewer of the “Person” and “Team Member” domains; comments in these domains came more often from peers and residents (“Team Member”) and patients, nurses and peers (“Person”). We hypothesize that this is because peers and residents work with students more closely than physicians, and that patients, nurses and peers relate more closely to students as people than do physicians. We suggest that in providing written comments, physicians focus more on the cognitive knowledge and skills associated with learning medicine (knowing, thinking, doing, reading, learning) and less on “softer” interpersonal behaviours (listening, explaining, helping, cooperating); it is possible that physicians find some elements of performance more legitimate to provide a written comment on than others.

Our findings suggest that important aspects of medical student performance can be observed by non-physician members of the surgical team. Some sub-domains were covered by multiple assessor groups, but in several areas comments were provided by only one group of assessors. These included listening & explaining (patients), physical examination (patients), organization of findings: diagnosis and management (residents), technical skills: gowning/gloving and asepsis (operating room nurses), follows instructions (operating room nurses) and leadership and resilience (peers). This observation suggests that each group of assessors brings something valuable to student assessment, and that omitting comments from one group would lead to the loss of observations from specific areas of student performance; as Lockyer and Clyman write, “additive value is accrued from comparison of multiple sources” [26]. While physicians could solicit comments from other assessor groups (patients, nurses, etc.), we believe that the method described here is more valid as it allows other members of the healthcare team to provide first-hand observations directly to students. We believe that including comments from other team members has the potential to improve student assessment by facilitating sampling from more domains of medical student performance [27,28].

Others have shown that non-physicians are able to evaluate the performance of physicians in training and practice [29-31], and have suggested that different assessor groups rate performance in different ways [32]. Several papers have suggested that assessment by nurses may yield different information than that obtained from physicians [28,33]. Peer assessment of medical students is also well-established and has been shown to improve student performance, especially interpersonal skills and professionalism [34-36]. Feedback from patients has also been shown to be useful, although most ratings and comments received are positive and complimentary, as we have observed [37-39]. Receiving feedback directly from patients is also likely to increase students’ awareness of the patients’ perspective on illness [40]. There is little work on the use of administrators in assessing medical students, but we believe that including their opinions is important as it is fairly simple to do and allows observations on issues such as absence, lateness and respect for non-medical staff. We also found that written comments provided by a range of assessors was a rich source of data for student assessment which proved helpful when making decisions on academic promotion and advancement in the months after the clerkship had finished.

We were pleasantly surprised at the quality and quantity of written comments provided to students, and hypothesize that the immediacy of the assessment model, with comments being written immediately after a period working with the assessor may have accounted for the large number of comments provided. Asking for ‘prompted comments’ in response to ‘Areas of Excellence’ and ‘Areas of Improvement’ may have helped assessors provide specific comments instead of more general observations [24]. We were also pleased with the number of comments received from the ‘non-traditional’ assessor groups such as peers, nurses and patients; together, these made up more than half of the comments received. We noted that the “valance” of written comments (the ratio of positive to negative) was 2,363:625, a ratio of 3.8:1. Others have reported valance ratios ranging from 2:1 to 15:1 [21,24,41].

We believe that the 4 main domains of the framework described here are distinct from one another and provide a useful way of considering medical student performance. The framework describes the clinical performance of medical students in more detail than previous work, and also provides more clarity on larger constructs such as ‘personality’ and ‘clinical skills’. It is also the first framework to consider comments from a range of assessor groups, and the first to suggest that a longer list of specific sub-domains can be grouped into four main areas of performance. The framework we have presented here has two potential applications. Firstly, it serves as a theoretical basis to explain what medical students do every day and how they are perceived by those with whom they work. Secondly, it has a practical application in helping to guide the written feedback which assessors provide to students; for instance, assessors could perhaps be reminded: “when commenting on a student’s performance, try to write down one thing from each of the four domains: Physician-in-Training, Learner, Team Member and Person”.

A number of other authors have also developed frameworks to describe the range of written comments given to medical trainees by physicians. Lye’s 2001 study of comments provided to students on a pediatrics clerkship identified a total of 12 domains of clinical performance, many of which are similar to those identified in our study [42]. Plymale *et al.* also studied comments given to students on a surgery clerkship and identified 21 domains of performance [8]. Sokol-Hessner identified 20 domains of performance in comments given to clerkship students [19], while Frohna *et al.* identified eight possible domains [41], and Schum six [24].

There are several arguments which support the validity of the framework developed in this study. Firstly, the number of comments received appeared to vary by the amount of time that each group of assessors would have been expected to spend with a student: thus, physicians, patients and peers spent the most time with the students, and left the most comments while administrators had the least interactions with the students and left the fewest comments. Secondly, there was evidence that the content of comments varied between assessors groups based on the expected context of the interaction with the student; in general, the assessor groups commented on what they would be expected to observe, and did not comment on areas they would not be expected to observe. Thus, operating room nurses commented on aseptic technique and not history-taking skills, while physicians commented on oral presentation skills but not helpfulness or cooperation. Patients did not comment on ‘Student as a Working Member of the Team’ at all, as they did not see them working in that context.

It is interesting to compare this framework to other frameworks such as CanMEDs [43]. The domain of ‘Physician-in-Training’ corresponds most closely with ‘Medical Expert’, while ‘Team Member’ aligns best with ‘Collaborator’. Other CanMEDs domains such as ‘Communicator’ and ‘Professional’ are represented in various sub-domains, while ‘Scholar’ and ‘Advocate’ were not strongly emphasized in the comments. It is interesting to note that much of the material coded under ‘Student as Person’ is not present in CanMEDs. A recent study describing a framework for written comments given to residents suggests that many written comments can be mapped onto CanMEDs domains, but that some comments fall outside the CanMEDs framework [44]. Two of the areas identified were ‘disposition (attitudes and personality)’ and ‘trajectory (norm reference, improvement and future predictions)’; we observed similar types of comments in this study, coded under ‘Student as Person’ and ‘Student as Learner: Improvement/Suitability’. While some may consider some of the sub-domains listed under ‘Person’ as intrinsic traits (eg. common sense, sense of humour), we believe that it is important to provide students with information about how their behaviour is perceived by their patients and those with whom they work, with the intention of helping them develop into more effective professionals.

This study has several limitations. The first is that it deals only with words written down to describe what was discussed at an encounter taking place to discuss performance which had already been observed. There is evidence that much of what is discussed in person is not recorded in written comments [19,45]; this concurs with anecdotal reports of the same in our program, and thus we surmise there may be elements of student performance which were not recorded. We hope that including comments from a variety of assessor groups ameliorates this effect to some degree. Secondly, we considered the possible interaction of the numerical items on the ratings form with the comments which were written down. It is possible that assessors would ‘take a cue’ to write a comment about an area of performance mentioned in the numerical items [41], or perhaps would not write a comment about a particular area of performance as a numerical rating had already been given. We did not detect any strong evidence of this; while many written comments corresponded with items on the ratings form, many written comments did not relate to any particular item. Thus, we could not entirely exclude this effect. Our study was limited in examining comments by administrators, as it included only one assessor of this type; we hope to conduct additional studies with a larger number of administrators in future. Lastly, our findings relate to the context of a hospital-based surgery clerkship; it is possible that different findings would be obtained if the study were repeated with different healthcare teams working in different settings.

We agree with other authors that written comments provided to students are a rich source of data [41]; we plan to continue to use and study this method of assessment, and to further validate the framework of comments we have developed. In future studies, we will compare written comments given to students in different clerkships, using comments given to individual students as the unit of analysis, and will also investigate the ways in which assessors decide on the content of the written comments they provide. We encourage others to apply the framework presented here in other settings to further refine our understanding of what medical students do and how it is perceived.

## Conclusions

In assessing the performance of medical students using written comments, it is important to consider “who writes what”. The study shows that written comments provided to medical students related to a wide range of observed student performance, and that different groups of assessors provide comments on different aspects of student performance. Comments provided from a single viewpoint may thus potentially under-represent or overlook some areas of student performance. The conceptual framework presented here may be useful in better understanding medical student performance, and in improving the content of written comments provided to students.

## Competing interests

The authors report no competing interests.

## Authors’ contributions

JW conceived of the study concept. NS was responsible for data collection. JW and NS were responsible for data analysis. JW and NS drafted the manuscript together and both approved the final manuscript.

## Authors’ information

Dr NS MSc EdD was Team Lead for Undergraduate Surgical Education in the Faculty of Medicine & Dentistry at the University of Alberta, Canada.Dr JW MB PhD FRCS (Gen Surg) MSc (Med Ed) is a consultant surgeon in Edmonton, Canada and is Senior Director of Undergraduate Surgical Education in the Faculty of Medicine & Dentistry at the University of Alberta, Canada.

## Pre-publication history

The pre-publication history for this paper can be accessed here:

http://www.biomedcentral.com/1472-6920/12/123/prepub

## References

[B1] Pelling S Kalen A Hammar M Wahlström O Preparation for becoming members of health care teams: findings from a 5-year evaluation of a student interprofessional training ward J Interprof Care 2011 25 328 332 10.3109/13561820.2011.578222 21635182

[B2] Farmer EA Beard JD Dauphinee WD LaDuca T Mann KV Assessing the performance of doctors in teams and systems Med Educ 2002 36 942 948 10.1046/j.1365-2923.2002.01311.x 12390462

[B3] Miller B Moore D Jr Stead W Beyond Flexner: a new model for continuous learning in the health professions Acad Med 2010 85 266 272 10.1097/ACM.0b013e3181c859fb 20107354

[B4] Harden RM Dent JA A Practical Guide for Medical Teachers 2009 Edinburgh: Churchill Livingstone

[B5] Norcini J Burch V Workplace-based assessment as an educational tool: AMEE Guide No. 31 Med Teach 2007 29 855 871 10.1080/01421590701775453 18158655

[B6] Williams RG Klamen DA McGaghie WC Cognitive, social and environmental sources of bias in clinical performance ratings Teach Learn Med 2003 15 270 292 10.1207/S15328015TLM1504_11 14612262

[B7] Bing You RG Greenberg LW Wiederman BL Smith CS A randomized multicenter trial to improve resident teaching with written feedback Teach Learn Med 1997 9 10 3 10.1080/10401339709539806

[B8] Plymale MA Donnelly MB Lawton J Pulito AR Mentzer RM Faculty evaluation of surgery clerkship students: important components of written comments Acad Med 2002 77 S45 S47 10.1097/00001888-200210001-00015 12377702

[B9] van Mook WNKA Gorter SL O'Sullivan H Wass V Schuwirth LW van der Vleuten CPM Approaches to professional behaviour assessment: Tools in the professionalism toolbox Eur J Int Med 2009 20 e153 e157 10.1016/j.ejim.2009.07.012 19892295

[B10] Green M Zick A Thomas JX Commentary: Accurate medical student performance evaluations and professionalism assessment: "Yes, we can!" Acad Med 2010 85 1105 1107 10.1097/ACM.0b013e3181e208c5 20592502

[B11] Rose M Widening the lens on standardized patient assessment: what the encounter can reveal about the development of clinical competence Acad Med 2001 76 856 859 10.1097/00001888-200108000-00023 11500293

[B12] Durning SJ Hanson J Gilliland W McManigle JM Waechter D Pangaro LN Using qualitative data from a program director's evaluation form as an outcome measurement for medical school Mil Med 2010 175 448 452 20572479 10.7205/milmed-d-09-00044

[B13] Burford B Illing J Kergon C Morrow G User perceptions of multi-source feedback tools for junior doctors Med Educ 2010 44 165 176 10.1111/j.1365-2923.2009.03565.x 20059677

[B14] Hoffman K Hosokawa M Donaldson J What criteria do faculty use when rating students as potential house officers? Med Teach 2009 31 e412 e417 10.1080/01421590802650100 19811177

[B15] Pulito AR Donnelly MB Plymale M Mentzer RM What do faculty observe of medical students’ clinical performance? Teach Learn Med 2006 18 99 104 10.1207/s15328015tlm1802_2 16626266

[B16] Hasley PB Arnold RM Summative evaluation on the hospital wards. What do faculty say to learners? Adv in Health Sci Educ 2008 14 431 439 10.1007/s10459-008-9127-1 18528775

[B17] Cohen GS Blumberg P Ryan NC Sullivan PL Do final grades reflect written qualitative evaluations of student performance? Teach Learn Med 1993 5 10 15 10.1080/10401339309539580

[B18] Mazor KM Canavan C Farrell M Margolis MJ Clauser BE Collecting validity evidence for an assessment of professionalism: findings from think-aloud interviews Acad Med 2008 83 S9 12 10.1097/ACM.0b013e318183e329 18820511

[B19] Sokol-Hessner L Shea JA Kogan JR The Open-Ended Comment Space for Action Plans on Core Clerkship Students’ Encounter Cards: What Gets Written? Acad Med 2010 85 S110 S114 20881692 10.1097/ACM.0b013e3181ed1c51

[B20] Littlefield JH DaRosa DA Paukert J Williams RG Klamen DL Schoolfield JD Improving resident performance assessment data: numeric precision and narrative specificity Acad Med 2005 80 489 495 10.1097/00001888-200505000-00018 15851464

[B21] Canavan C Holtman MC Richmond M Katsufrakis PJ The Quality of Written Comments on Professional Behaviors in a Developmental Multisource Feedback Program Acad Med 2010 85 S106 S109 20881691 10.1097/ACM.0b013e3181ed4cdb

[B22] White J Sharma N Using multi-source feedback to assess medical students learning on an interprofessional surgical healthcare team Proceedings of the 14th Ottawa Conference on Assessment in the Healthcare Professions 2010 368 369

[B23] Sharma N Cui Y Leighton J White J Team-based assessment of medical students in a clinical clerkship is feasible and acceptable Med Teach 2012 34 7 555 561 10.3109/0142159X.2012.669083 22746962

[B24] Schum TR Krippendorf RL Biernat KA Simple feedback notes enhance specificity of feedback to learners Ambul Pediatr 2003 3 9 11 10.1367/1539-4409(2003)003<0009:SFNESO>2.0.CO;2 12540246

[B25] White MD Marsh EE Content analysis: A flexible methodology Library Trends 2006 55 22 45 10.1353/lib.2006.0053

[B26] Lockyer JM L Clyman SG Holmboe ES, Hawkins RE M Multisource feedback (360-degree evaluation) Practical Guide to the Evaluation of Clinical Competence 2008 1 75 85

[B27] Ginsburg S Regehr G Hatala R McNaughton N Frohna A Hodges B Context, conflict, and resolution: a new conceptual framework for evaluating professionalism Acad Med 2000 75 S6 S11 10.1097/00001888-200010001-00003 11031159

[B28] Johnson D Cujec B Comparison of self, nurse, and physician assessment of residents rotating through an intensive care unit Crit Care Med 1998 26 1811 1816 10.1097/00003246-199811000-00020 9824072

[B29] Wenrich MD Carline JD Giles LM Ramsey PG Ratings of the performances of practicing internists by hospital-based registered nurses Acad Med 1993 68 680 687 10.1097/00001888-199309000-00014 8397633

[B30] Kaplan CB Centor RM The use of nurses to evaluate house officers' humanistic behavior J Gen Intern Med 1990 5 410 414 10.1007/BF02599428 2231037

[B31] Whitehouse A Hassell A Bullock A Wood L Wall D 360 degree assessment (multisource feedback) of UK trainee doctors: Field testing of team assessment of behaviours (TAB) Med Teach 2007 29 171 176 10.1080/01421590701302951 17701629

[B32] Bullock AD Hassell A Markham WA Wall DW Whitehouse AB How ratings vary by staff group in multi-source feedback assessment of junior doctors Med Educ 2009 43 516 520 10.1111/j.1365-2923.2009.03333.x 19493174

[B33] Ogunyemi D Gonzalez G Fong A Alexander C Finke D Donnon T From the eye of the nurses: 360-degree evaluation of residents J Contin Educ Health Prof 2009 29 105 110 10.1002/chp.20019 19530193

[B34] Nofziger AC Naumburg EH Davis BJ Mooney CJ Epstein RM Impact of peer assessment on the professional development of medical students: a qualitative study Acad Med 2010 85 140 147 10.1097/ACM.0b013e3181c47a5b 20042840

[B35] Finn GM Garner J Twelve tips for implementing a successful peer assessment Med Teach 2011 33 443 446 10.3109/0142159X.2010.546909 21355687

[B36] Cushing A Abbott S Lothian D Hall A Westwood OMR Peer feedback as an aid to learning – What do we want? Feedback. When do we want it? Now! Med Teach 2011 33 e105 e112 10.3109/0142159X.2011.542522 21275532

[B37] Lyons O Willcock H Rees J Let the patient teach: patient feedback will help prepare medical students for the changing healthcare world Clin Teach 2009 34 256 10.3109/0142159X.2012.652708 22364466

[B38] Burford B Bedi A Morrow G Kergon C Collecting patient feedback in different clinical settings: problems and solutions Clin Teach 2009 6 259 264 10.1111/j.1743-498X.2009.00316_1.x

[B39] Braend AM Gran SF Frich JC Lindbaek M Medical students’ clinical performance in general practice Triangulating assessments from patients, teachers and students Med Teach 2010 32 333 339 10.3109/01421590903516866 20353331

[B40] Gran SF Braend AM Lindbaek M Triangulation of written assessments from patients, teachers and students: Useful for students and teachers? Med Teach 2010 32 e552 e558 10.3109/0142159X.2010.528808 21090943

[B41] Frohna A Stern D The nature of qualitative comments in evaluating professionalism Med Educ 2005 39 763 768 10.1111/j.1365-2929.2005.02234.x 16048618

[B42] Lye PS Biernat KA Bragg DS Simpson DE A pleasure to work with–an analysis of written comments on student evaluations Ambul Pediatr 2001 1 128 131 10.1367/1539-4409(2001)001<0128:APTWWA>2.0.CO;2 11888388

[B43] Frank JRE The CanMEDS 2005 physician competency framework. Better standards. Better physicians. Better care 2005 Ottawa: The Royal College of Physicians and Surgeons of Canada

[B44] Ginsburg S Gold W Cavalcanti RB Kurabi B McDonald-Blumer H Competencies plus: the nature of written comments on internal medicine residents' evaluation forms Acad Med 2011 86 S30 S34 21955764 10.1097/ACM.0b013e31822a6d92

[B45] Hemmer PA Hawkins R Jackson JL Pangaro LN Assessing how well three evaluation methods detect deficiencies in medical students' professionalism in two settings of an internal medicine clerkship Acad Med 2000 75 167 173 10.1097/00001888-200002000-00016 10693850

